# Recent advances in neoadjuvant therapy for breast cancer

**DOI:** 10.12703/r/10-2

**Published:** 2021-01-04

**Authors:** David A Potter, César A Herrera-Ponzanelli, Diego Hinojosa, Rafael Castillo, Irwin Hernandez-Cruz, Victor A Arrieta, Michael J Franklin, Douglas Yee

**Affiliations:** 1 University of Minnesota, Minneapolis, MN, USA; 2 Unidad de Investigación Biomédica en Cáncer, Instituto Nacional de Cancerología-Instituto de Investigaciones Biomédicas, UNAM, México; 3 Department of Neurosurgery, Northwestern University Feinberg School of Medicine, Chicago, IL, USA; 4 PECEM, Facultad de Medicina, Universidad Nacional Autónoma de México, México City, México

**Keywords:** breast cancer, neoadjuvant therapy, adaptive design

## Abstract

Neoadjuvant trials for early breast cancer have accelerated the identification of novel active agents, enabling streamlined conduct of registration trials with fewer subjects. Measurement of neoadjuvant drug effects has also enabled the identification of patients with high risk of distant recurrence and has justified development of additional adjuvant approaches to improve outcomes. Neoadjuvant evaluation of new drugs was significantly improved by the introduction of pathologic complete response (pCR) rate as a quantitative surrogate endpoint for distant disease-free survival (DDFS) and event free survival (EFS). The neoadjuvant phase 2 platform trial I-SPY 2 simultaneously tests multiple drugs across multiple breast cancer subtypes using Bayesian methods of adaptive randomization for assessment of drug efficacy. In addition to the pCR endpoint, the I-SPY 2 trial has demonstrated that the residual cancer burden (RCB) score measures gradations of tumor response that correlate with DDFS and EFS across treatments and subtypes. For HER2-positive and triple-negative breast cancers that have failed to attain pCR with neoadjuvant chemotherapy (NAC), effective modifications of adjuvant treatment have improved outcomes and changed the standard of care for these subtypes. Neoadjuvant therapy is therefore preferred for stage II and III, as well as some stage I, HER2-positive and triple-negative tumors. Neoadjuvant endocrine therapy (NET) strategies have also emerged from innovative trials for stage II and III estrogen receptor (ER)-positive/HER2-negative tumors, as in the ALTERNATE trial. From neoadjuvant trials, opportunities have emerged to de-escalate therapy on the basis of metrics of response to chemotherapy or hormonal therapy. Neoadjuvant therapy for early breast cancer is therefore emerging as a promising approach to accelerate new drug development, optimize treatment strategies, and (where appropriate) de-escalate neoadjuvant therapy.

## Purpose of this review

The purpose of this review is to critically evaluate the results of trials of neoadjuvant chemotherapy (NAC) and neoadjuvant endocrine therapy (NET) for different breast cancer subtypes, discuss how neoadjuvant responses can inform adjuvant therapy, and highlight the importance of pathological complete response (pCR) as a surrogate marker of distant disease-free survival (DDFS) (the length of survival time following primary treatment with no distant recurrence of cancer), distant recurrence free survival (DRFS) (survival time from surgery to first distant recurrence), and event free survival (EFS) which is interchangeable with disease-free survival (DFS) (the length of time after primary treatment that the patient is free of complications or events related to the cancer), in the age of adaptive trial design. Notably, in contrast to overall survival (OS), precise definitions of DFS and similar, related endpoints vary somewhat from trial to trial. pCR is generally a composite endpoint of primary tumor and lymph nodes. While pCR correlates with lower risk of recurrence, risk of recurrence still occurs and reduction of this risk remains an important goal. We also highlight functional and genomic predictive biomarkers in NET and their prediction of recurrence free survival (RFS), essentially equivalent to EFS.

## Introduction

Administration of NAC before surgery has several advantages that are not afforded by adjuvant chemotherapy after surgery. These advantages include the ability to determine response to therapy during treatment, which may open avenues for further risk reduction by subsequent adjuvant therapy, the development of imaging biomarkers for drug response^1–6^, the study of mechanisms of treatment resistance^7,8^, and the development of new strategies to employ novel agents for cancer treatment across breast cancer subtypes^9,10^. By initiating therapy with neoadjuvant treatment, clinicians allow time for assessment of germline genetic risk factors, determination of the most appropriate surgery and potential reconstruction, and choice of neoadjuvant treatment based on risk stratification. Furthermore, NAC could frequently downstage the primary tumor and lymph nodes, allowing conversion of the planned surgery from a mastectomy to a lumpectomy, allowing for breast conservation^11^, and potentially allowing omission of an axillary lymph node dissection^12^. Breast conservation is made more likely by neoadjuvant therapy in HER2-positive and triple-negative tumors, subtypes which showed the highest rates of clinically meaningful tumor reduction in the Investigation of Serial studies to Predict Your Therapeutic Response with Imaging and molecular AnaLysis 1 (I-SPY 1) trial^11^. Neoadjuvant hormonal therapy has also increased breast conservation rates in estrogen receptor (ER)-positive/HER2-negative breast cancer and will be discussed below^13^.

Despite these potential advantages, the first step in the development of NAC was to establish its safety, meaning that administration of chemotherapy before surgery was non-inferior in terms of long-term outcomes, including DFS and OS. The safety and efficacy of NAC were demonstrated through National Surgical Adjuvant Breast and Bowel Project (NSABP) clinical trials B-18 and B-27^14–17^. The NSABP B-18 and B-27 clinical trials provided a strong rationale for this approach, showing that presurgical chemotherapy resulted in similar DFS as postsurgical treatment^16,17^. The discovery of DFS equivalency allowed for the capture of pathologic, biologic, and radiographic surrogate markers of tumor response to neoadjuvant therapy that was previously unattainable, thereby opening new vistas for testing of novel anticancer drugs for efficacy before surgery. These approaches were developed in the innovative I-SPY 1 neoadjuvant trial (see below)^18,19^, the series of German Breast Group neoadjuvant trials^20^, and in trials developed by many other investigators. Furthermore, platform neoadjuvant trials incorporating magnetic resonance imaging (MRI), such as I-SPY 1 and its successor I-SPY 2, provide an opportunity to not only increase efficacy of treatment but develop new approaches to avoid overtreatment.

## Rationale for neoadjuvant therapy: the NSABP B-18 and B-27 trials and association of pCR with DFS

The NSABP B-18 trial was designed to determine whether preoperative treatment with four cycles of doxorubicin/cyclophosphamide (AC) would improve overall survival (OS) and DFS when compared with the same treatment given postoperatively. At 9 years of follow-up, there was no difference in survival or DFS between the two groups^14,15^. A critical observation during this follow-up was that pCR correlated with OS, and this finding became stronger with longer follow up^21^. Primary tumor response graded as pCR, pINV (pathologic non-responder with residual cancer), clinical partial response (cPR), or clinical non-responder (cNR) and was associated with outcome measures of OS (*P* = 0.0008), DFS (*P* = 0.00005), and RFS (*P* = 0.0002)^21^. The B-18 trial established that chemotherapy can be given before surgery with no loss of efficacy, opening the way to measuring chemotherapy effect and the testing of novel agents, as in the B-27 trial.

Critically important for the development of NAC for breast cancer was the confirmation that the outcomes of neoadjuvant and adjuvant chemotherapy were similar in the NSABP B-27 trial and that achievement of pCR correlated with DFS and OS^16^. Preoperative addition of the then-novel docetaxel (T) to AC in the B-27 trial improved DFS and this was associated with a doubling of the pCR rate. Furthermore, pCR was prognostic of OS regardless of treatment (HR = 0.33, 95% confidence interval [CI] 0.23–0.47; *P* <0.0001)^16^. This relationship between pCR and outcomes was consistent with prior results of the NSABP B-18 trial, which also showed that clinical and pathologic tumor response was prognostic of OS, DFS, and RFS^14,15,21^. A combined analysis of the long-term outcomes of the B-18 and B-27 trials confirmed the association of pCR and DFS, which showed similar correlations (B-18 HR = 0.32; *P* <0.0001 and B-27 HR = 0.36; *P* <0.0001)^17^. While ER-negative tumors in the B-27 trial had a higher pCR rate in response to neoadjuvant therapy compared with ER-positive tumors, docetaxel improved pCR rates of ER-negative and ER-positive tumors, indicating the feasibility of using a novel agent to improve pCR regardless of ER status^22^. This observation supported the idea of the platform trial testing new drugs across different histologies, when appropriate. Furthermore, from the B-18 and B-27 trials, the rate of pCR became a valuable tool as an early indicator of neoadjuvant drug efficacy and as a surrogate for DFS.

The approach of NAC was further supported by the CTNeoBC meta-analysis, in which results of 12 international NAC trials were pooled to study the relationship between chemotherapy response and long-term outcomes^23^. Complete response in breast and lymph nodes (ypT0ypN0 or ypT0/is ypN0) was associated with improved EFS and OS compared with complete response in the breast alone (ypT0/is)^23^. This meta-analysis also showed that the association between pCR and EFS was strongest in patients with triple-negative breast cancer (TNBC) and in patients with HER2-positive, hormone receptor–negative tumors who received trastuzumab^23^. This meta-analysis provided additional support for the concept of a composite endpoint of tumor and lymph nodes for assessment of NAC, which continues to be used in neoadjuvant trials. Together, this meta-analysis provided a strong rationale to use pCR rate as an endpoint for neoadjuvant clinical trials of novel agents, which could accelerate new drug development. It must be noted that these neoadjuvant trials included ER-positive tumors and were performed prior to the incorporation of gene expression profiling in neoadjuvant trial design. Since the majority of ER-positive tumors receive no benefit from chemotherapy as demonstrated by the TAILORx and MINDACT trials^24,25^, the inclusion of these “chemotherapy-resistant” tumors in chemotherapy trials weakens the association between NAC and long-term outcomes, as endocrine therapy is responsible for improvement of long-term outcomes. Notably, I-SPY 2 excludes most clinical high-risk, genomic low-risk (70-gene assay)^25^ patients from receiving chemotherapy, although HER2-positive and ER-negative genomic low-risk patients are allowed.

Further supporting the safety of NAC, a meta-analysis of long-term outcomes of 10 trials of neoadjuvant versus postoperative chemotherapy (performed by the Early Breast Cancer Trialists’ Collaborative Group) showed no difference in distant recurrence, breast cancer mortality, or death from any cause^26^. There was increased frequency of breast-conserving therapy with neoadjuvant (65%) versus adjuvant (49%) chemotherapy^26^. While this meta-analysis found that NAC correlated with higher local recurrence (21.4%) compared with adjuvant chemotherapy (15.9%) (*P* = 0.0001)^26^, this meta-analysis included only one trial where neoadjuvant taxane was used^27^, in contrast to trials such as NSABP B-27, I-SPY 1, and I-SPY 2 (see below), where neoadjuvant taxanes were used. Furthermore, the design of the I-SPY 1 and I-SPY 2 trials incorporated MRI before surgery, which may impact rate of local recurrence^9,10,18,19^. Neoadjuvant compared to adjuvant chemotherapy showed no difference in distance recurrence (15 year risk 38.2 vs. 38.0%; rate ratio 1.02, 95% CI 0.92–1.14; *P* = 0.66), breast cancer mortality (34.4 vs. 33.7%; rate ratio 1.06, 95% CI 0.95–1.18; *P* = 0.31), or death from any cause (40.9 vs. 41.2%; rate ratio 1.04, 95% CI 0.94–1.15; *P* = 0.45)^26^.

## Residual cancer burden after neoadjuvant therapy: development of a prognostic tool

Although pCR was developed as an endpoint in the B-18 and B-27 trials, Symmans *et al*. recognized that while no tumor in the tumor bed after NAC was a clear endpoint (pT0), the presence of residual nodal metastases, minimal residual disease in the tumor bed, and residual ductal carcinoma *in situ* (DCIS) was not well defined^28^. A large single-institution collection of development and validation tumor specimens was available for analysis of these questions (MD Anderson Cancer Center, Houston, TX, USA) and permitted the development of the residual cancer burden (RCB) index^28^. In its original inception, RCB was based on the observation that patients receiving NAC for early breast cancer (stage 1–III) who attained a pCR had better outcomes in terms of DDFS^28^. Quantifying the response to see whether there was a relationship between residual cancer and clinical outcomes was therefore a goal. The RCB was developed to accomplish this goal, as a continuous index combining measurements of the primary tumor, in terms of size and cellularity, and nodal metastases evaluated in terms of number and size. The RCB is therefore a quantitative composite measure of outcome. Correlation of DDFS with RCB was modeled using a multivariate Cox regression model^28^. Prospective validation of RCB stratification was then performed^29^. Most notably, with long-term follow-up of patients who received neoadjuvant therapy, tailored to breast cancer subtype, the 10-year RFS rates were defined for four classes of RCB (RCB-0 = pCR in tumor and nodes or varying degrees of residual tumor classified as RCB-1, RCB-2, or RCB-3)^29^. The RCB scores across the four categories from 0 to 3 correlated with RFS values of 86%, 81%, 55%, and 23% in the triple-negative subtype; 83%, 97%, 74%, and 52% in the hormone receptor–positive/HER2-negative subtype; and 95%, 77%, 47%, and 21% in the HER2-positive subtype^29^. The RCB score was prognostic of RFS in all three subtypes, independent of other clinical and pathologic variables, using standard chemotherapy platforms for these subtypes. Having demonstrated utility for measuring response to standard chemotherapy, RCB evaluation was further used to assess outcomes of novel agents in combination with a standard NAC backbone in the I-SPY 2 clinical trial of high-risk breast cancers across a range of subtypes. In the I-SPY 2 trial, the RCB score was strongly and independently prognostic in all breast cancer subtypes^30^. An additional take home is that because RCB is a composite endpoint after neoadjuvant therapy, sentinel node and axillary surgery (if indicated) should occur after neoadjuvant therapy^12,31^.

## ALTTO versus neo-ALTTO: the significance of pCR

The development of the HER2 tyrosine kinase inhibitor lapatinib provided an opportunity to compare the efficacy of a new targeted agent in the neoadjuvant^32,33^ versus adjuvant^34^ settings by asking whether the pCR rate in the neoadjuvant neo-ALTTO trial^32^ predicted a significant DFS in the adjuvant ALTTO trial^34^. Lapatinib was associated with clinical activity in both trials, but the neoadjuvant trial was deemed “significant” and the adjuvant trial “not significant” on the basis of statistical design^35^. In neo-ALTTO, the pCR rate was higher with lapatinib and trastuzumab (51.3%; 95% CI 43.1–59.5) compared to trastuzumab (29.5%; 95% CI 22.4–37.5), the difference being 21.1% (9.1–34.2, *P* = 0.0001)^32^. The DFS in the ALTTO trial did not reach statistical significance (HR 0.84, 97.5% CI 0.70–1.02; *P* = 0.048)^34^. The ALTTO trial did not reach significance likely because it included a test of non-inferiority of trastuzumab (12 weeks) followed after washout by lapatinib (34 weeks) vs. trastuzumab control (52 weeks) performed in addition to comparison of lapatinib (52 weeks) and lapatinib and trastuzumab (52 weeks). In a subsequent meta-analysis relating pCR and subsequent EFS in six neoadjuvant trials, including neo-ALTTO, the HR for EFS in patients achieving a pCR versus not was 0.39 (95% CI 0.31–0.50)^35^. There was some debate because the improved pCR rate seen in the neo-ALTTO trial did not appear to predict improved EFS (interchangeable with DFS) in the “confirmatory” ALTTO study, but a strong counterargument was made that the neo-ALTTO results actually predicted EFS in ALTTO when appropriate statistical modeling was employed^35^. Also, the therapy in ALTTO was not precisely the same as with neo-ALTTO, which may have reduced the effect in ALTTO. Thus, the results of the two trials were in descriptive agreement with each other. An advantage of the neoadjuvant approach is the acceleration of drug development by identifying the right drugs for the right subtypes, which may exhibit large effect differences, thereby reducing the sample size and the time required to conduct a definitive phase 3 trial of appropriate design^35^.

## I-SPY 1 trial: correlation of pCR with RFS across breast cancer subtypes and feasibility of imaging biomarker discovery

The I-SPY 1 (CALGB 150007/150012, ACRIN 6657) trial was an integrated conducted by 9 cooperating institutions. I-SPY 1 was a trial to test the feasibility of integrating multiple modalities for evaluation of response to NAC^18^. The utility of pCR as a predictor of DFS, shown in the B-18 and B-27 trials and others, led to this trial, which tested the integration of multiple modalities of assessment of treatment response in the neoadjuvant setting, including biopsy, MRI, a 70-gene assay assessing outcome risk (MammaPrint; Agendia, Irvine, CA, USA)^36^, and measurement of chemotherapy effectiveness by rate of pCR^18,19,37^. The I-SPY 1 trial played a key role in the validation of MRI and tumor biopsies at intermediate endpoints for breast cancer biomarker development and pCR as a biomarker for treatment efficacy^18,19,37^. Similar to RCB, with pCR as the endpoint axillary surgery must occur after neoadjuvant therapy, and this is the methodology used in the subsequent I-SPY 2 trial as well^12,31^. I-SPY 1 showed correlation between both RCB and pCR and RFS outcomes^37,38^, as did I-SPY 2 which came later. pCR was found to correlate with RFS within each established breast cancer subset by multivariate analysis^37^. By multivariate analysis, molecular signatures, including the 70-gene signature, added to clinical demographics and pCR aided in predicting RFS^19^. Furthermore, MRI tumor assessment during NAC was found to be a strong predictor of pCR and volumetric tumor reduction early in NAC was the strongest MRI-derived predictor of pCR^39^. The I-SPY 1 trial therefore provided an accelerated platform to test targeted agents for efficacy in specific molecular subgroups, for the purpose of improving pCR rates.

## I-SPY 2 trial: a Bayesian adaptive randomization phase 2 platform trial

Using the framework developed in the I-SPY 1 clinical trial, the ongoing Bayesian adaptive design phase 2 platform trial called I-SPY 2 was initiated in 2011 as a collaboration between academic investigators, the NCI, US FDA, and the pharmaceutical and biotechnology industries under the auspices of the NIH Biomarkers Consortium^40^. This trial was designed to simultaneously compare the efficacy of multiple novel drugs in combination with a standard platform while not directly comparing individual drugs with each other. This concept allows efficient throughput of new drugs. Key to the design of the I-SPY 2 clinical trial was the continuous assessment of pCR, which enabled the adaptive design of the trial to change randomization during active enrollment based on pCR. This design eliminates drugs that are underperforming early and promotes drugs that are performing well. The patient population chosen for the trial has stage II or III breast cancer with a tumor size of at least 2.5 cm and with stratification by nodal status, hormone receptor status, HER2 status, and the 70-gene MammaPrint (Agendia) assay^9,10,36,41^. MammaPrint high-risk patients are eligible regardless of ER or HER2 status, whereas eligible MammaPrint low-risk patients are eligible if they are HER2-positive or ER-negative. Notably, the MammaPrint predicts response to adjuvant chemotherapy or NAC across breast cancer subtypes and is also prognostic in terms of DDFS^42^. Patients eligible for randomization are then recategorized according to subclassifying biomarker profiles. Patients are randomly assigned to a control or treatment arm on the basis of real-time assessment of pCR across the arms of the study, and this process is described as a “randomization engine”. A major achievement of this study was the organizational infrastructure required to recruit and follow sufficient numbers of patients for the trial design, which has involved more than 20 institutions.

For HER2-negative breast cancer, the control arms generally consist of weekly paclitaxel for 12 weeks followed by dose-dense (every 2 weeks with growth factor support) AC (ddAC) for four cycles. Novel agents for HER2-negative breast cancer have been generally tested in combination with paclitaxel. However, there have been exceptions; for example, for HER2-negative breast cancer, veliparib was tested in combination with carboplatin in addition to paclitaxel^10^, and talazoparib was tested in combination with irinotecan without paclitaxel. For HER2-positive breast cancer, the control arm initially consisted of trastuzumab and paclitaxel, but later pertuzumab was added to trastuzumab. The novel agent neratinib was tested in combination with paclitaxel and compared to the trastuzumab and paclitaxel control arm in the HER2-positive signature. Neratinib was also tested in combination with paclitaxel in HER2-negative breast cancer while the control arm was paclitaxel^9^. Pertuzumab was tested in combination with trastuzumab and paclitaxel and compared to a control arm of trastuzumab and paclitaxel in the HER2-positive signature. After graduation, pertuzumab/trastuzumab/paclitaxel became the control arm for HER2-positive breast cancer. When trastuzumab emtansine (T-DM1) was tested with pertuzumab, paclitaxel was deleted.

Each investigational agent is assigned a biomarker signature determining which patients would be randomly assigned to that agent, based on a biological hypothesis of agent activity. Adaptive randomization is performed using blinded real-time measurement of pCR status of patients who received novel agents A, B, C, and so on in separate arms of the trial. The pCR information is used for real-time assessment of likelihood of success or failure of a given agent in a simulated subsequent phase 3 clinical trial of 300 patients randomly assigned 1:1 comparing the novel agent with a control arm. Futility is determined to be a predicted probability of success of less than 10% across the biomarker signatures^9,10^. Graduation of a drug from I-SPY 2 means an 85% Bayesian-predicted probability of success using pCR as an endpoint in the 1:1 randomly assigned 300-patient trial^9,10^. Notably, the testing of neratinib in the HER2-negative arms was stopped by the adaptive randomization algorithm due to futility, measured by pCR^9^.

The I-SPY 2 trial integrates two interlocking concepts: the idea that statistical inference from pCR data using Bayes’ theorem may be used in real time to update the probability that a drug could potentially demonstrate meaningful activity in a phase 3 trial and the idea that multiple drugs may be compared simultaneously with a single standard-of-care chemotherapy regimen but not directly to each other. This approach combines a practical endpoint with efficient throughput. Furthermore, a screen is applied to identify high-risk patients and assign them by biomarker signatures, thereby getting the right drugs to the right patients. This concept therefore appeals to biopharma companies because drugs without a strong effect on pCR fail early and more effective drugs succeed quickly.

From a patient perspective, the phase 2 design is appealing because all patients receive an effective platform regimen while some receive the addition of a novel drug assigned by tumor biomarker. In either case, patients are contributing to novel drug development in an environment in which all receive safe and effective therapy and some may receive an additional experimental agent with potentially added value. It is important to note that the real-time pCR data on which drug assignment is based are blinded to the investigators and patients and that the assignment is algorithm-driven. Whereas the assigned regimen is known, the reason the patient received the assigned regimen is not known. Bayesian adaptive randomization allows minimization of the number of patients who need to be randomly assigned for a subsequent phase 3 trial that is designed to compare the experimental regimen versus standard of care. The adaptive design concept was a natural consequence of these goals, which aligned with the multiple stakeholders: breast cancer patients who want cures, their physicians who seek to provide the best treatment for their patients while advancing knowledge about new options, and the biopharma companies that seek to develop novel agents. The common pivot on which this enterprise was based is the pCR and RCB determined by pathologists, whose workflow is integrated in real time with the adaptive trial design. The concept of a master platform trial was born, allowing tailoring of the right drug to the right disease subtype.

## I-SPY 2 trial: primary endpoints and secondary objectives

The primary endpoints of the I-SPY 2 trial are to determine whether adding an investigational agent (or agents) to neoadjuvant paclitaxel (with or without trastuzumab) and AC increases the probability of pCR over standard NAC alone for each of the biomarker signatures established at trial entry and to determine, for each investigational agent used, the predictive probability of success in a subsequent phase 3 trial for each possible biomarker signature. Probability of pCR is defined as probability of no residual invasive cancer in the tumor bed or lymph nodes (ypT0 and ypN0) in the intent-to-treat population. NAC has two functional roles for the patient: to reduce the size of the primary tumor, facilitating breast-conserving surgery^14,15^, and to reduce or eliminate micrometastatic disease that may have spread early on to sites distant from the breast^43^. NAC is expected to inhibit tumor cell seeding, survival, and growth in the primary and micrometastatic settings. Attainment of pCR therefore is expected to correlate with improved DFS/EFS.

A secondary objective of the I-SPY 2 trial was to build predictive and prognostic indices based on qualification biomarkers to predict pCR and RCB. It was hypothesized that quantitation of tumor response by RCB could be useful in predicting clinical outcomes^28^. It was natural to test RCB in the framework of the I-SPY 2 clinical trial as a predictor of agent efficacy as measured by EFS and DRFS. When the effect of pembrolizumab was measured in the I-SPY 2 trial, this agent was associated with a lower RCB for each cohort evaluated: hormone receptor–positive/*ERBB2* (formerly HER2)-negative, *ERBB2*-negative, and TNBC^44^. When 3-year outcomes of EFS and DRFS were studied across the I-SPY 2 trial incorporating data from nine novel agents and 950 participants, of whom 330 achieved pCR, the HRs for pCR versus non-pCR were 0.19 for EFS (95% CI 0.12–0.31) and 0.21 for DRFS (95% CI 0.13–0.34) and were similar across molecular subtypes, ranging from 0.14 to 0.18 for EFS and 0.01 to 0.20 for DRFS^45^. A comprehensive meta-analysis of neoadjuvant trials, including I-SPY 1, but not I-SPY 2, found a similar correlation between pCR and DDFS, particularly in TNBC and HER2-positive breast cancer^46^.

## I-SPY 2 trial: agents graduated and association of RCB and outcomes

Drugs that have graduated from the I-SPY 2 clinical trial have included the tyrosine kinase inhibitor neratinib in the HER2-positive, hormone receptor negative signature^9^, the PARP inhibitor veliparib with carboplatin (HER2-negative subgroups)^10^, the antibody–drug conjugate (ADC) T-DM1 (HER2-positive subgroups compared with paclitaxel/trastuzumab), the antibody pertuzumab when added to paclitaxel/trastuzumab, the PD-1 antibody pembrolizumab (HER2-negative)^44^, and the pan-AKT inhibitor MK-2206 (HER2-positive and TNBC subtypes)^47^. The subsequent phase 3 BrighTNess trial showed that whereas the addition of veliparib and carboplatin to neoadjuvant paclitaxel followed by AC in TNBC increased the attainment of pCR relative to paclitaxel alone, the addition of veliparib to carboplatin and paclitaxel did not increase pCR relative to carboplatin and paclitaxel^48^. This result did show the potential value of carboplatin in neoadjuvant therapy of TNBC. Addition of carboplatin to paclitaxel did increase toxicity, but the toxicities were manageable^48^. The KEYNOTE-522 trial provided phase 3 confirmation of neoadjuvant efficacy of pembrolizumab in the TNBC signature, predicted by I-SPY 2^49^. The combination of durvalumab/olaparib/paclitaxel also recently graduated from I-SPY 2 in the ER-positive/HER2-negative (MammaPrint ultrahigh-MP2) and TNBC subtypes^50^.

## Rationale for neoadjuvant therapy as a standard of care for patients with triple-negative breast cancer

A strong rationale for use of NAC for patients with TNBC—based on efficacy, safety, and prognostic information—is provided by the recent phase 3 trial KEYNOTE-522, which tested the benefit of pembrolizumab versus placebo in combination with carboplatin and paclitaxel followed by pembrolizumab versus placebo in combination with every-3-week AC or epirubicin/cyclophosphamide (EC) in patients with stage II or III TNBC^49^. Pembrolizumab or placebo was continued after surgery for 27 weeks. The first interim analysis showed that pembrolizumab treatment resulted in a significant improvement of pCR rates, regardless of PD-L1 combined positive score^49^. The pCR rate was 64.8% in the pembrolizumab–chemotherapy group (95% CI, 59.9–69.5) and 51.2% (95% CI, 44.1–58.3) in the placebo–chemotherapy group (estimated treatment difference, 13.6 percentage points; 95% CI, 5.4–21.8; *P* <0.001). For patients with PD-LI–positive tumors, the pCR rate was 68.9% with pembrolizumab versus 54.9% with placebo and chemotherapy^49^. For those with PD-L1–negative tumors, the pCR rate was 43.3% with pembrolizumab versus 30.3% with placebo. After 15.5 months of median follow up, 7.4% of patients in the pembrolizumab–chemotherapy group and 11.8% in the placebo–chemotherapy group had disease progression that could not be addressed by surgery, local or distant recurrence, or a second primary tumor, or died from any cause (hazard ratio, 0.63; 95% CI, 0.43–0.93)^49^. In terms of toxicity, 12.9% of pembrolizumab-treated patients had grade >3 immune related adverse events of special interest compared to 1.8% on the placebo arm. Immune related events included hypothyroidism, skin reactions, hyperthyroidism, adrenal insufficiency, pneumonitis, colitis, hypophysitis, thyroiditis, and hepatitis^49^. While EFS and OS remain to be reported for KEYNOTE-522, a large prior meta-analysis^23^ showed strong correlation between pCR and EFS as well as OS. Furthermore, the I-SPY 2 trial showed, across multiple neoadjuvant regimens, a 3-year EFS and DRFS of 95% for patients who achieved pCR^45^. The KEYNOTE-522 trial therefore provides a strong rationale for neoadjuvant pembrolizumab in combination with carboplatin, paclitaxel followed by pembrolizumab in combination with anthracycline/cyclophosphamide, followed by 27 weeks of adjuvant pembrolizumab for stage II and III TNBC. What is not known is the contribution of additional adjuvant pembrolizumab. Another unknown is the contribution of carboplatin in this trial. The use of adjuvant or neoadjuvant carboplatin results in increased toxicity across multiple trials and its use should be informed by a risk–benefit analysis of the literature^51^. Moreover, published regimens with acceptable toxicity should be adhered to.

Notably, two randomized neoadjuvant trials of PD-L1 antibodies, NeoTRIPaPDL1 which tested atezolizumab and chemotherapy (NCT02620280)^52^ and GeparNuevo which tested durvalumab and chemotherapy in TNBC^53^, did not show increase in pCR with addition of this PD-L1 antibody compared to placebo. The GeparNuevo trial did show a significant increase in pCR in a subset of patients who received a window of 2 weeks of durvalumab before beginning chemotherapy (pCR 61.0% versus 41.4%, OR = 2.22, 95% CI 1.06–4.64, *P* = 0.035)^53^.

A further rationale for NAC for TNBC is that subsequent adjuvant therapy can be implemented with the goal of improving outcomes for patients who lack a pCR result. The CREATE-X and GEICAM trials tested these approaches (see below).

## The CREATE-X and related TNBC trials of capecitabine strategies

Further rationale for starting treatment of TNBC with NAC is provided by the recent discovery of effective adjuvant chemotherapy for patients with HER2-negative breast cancer who fail to achieve pCR^54^. Across breast cancer subtypes, patients who have residual disease after NAC have a worse prognosis than patients who attain a pCR^17,23,28,29,33,45,55–58^. This concern provided a rationale for the phase 3 CREATE-X trial (UMIN000000843), which tested whether adjuvant capecitabine improves OS for patients with HER2-negative breast cancer who failed to attain pCR with NAC^54^. In the CREATE-X trial, patients were randomly assigned in a 1:1 unblinded fashion to adjuvant capecitabine for 18 or 24 weeks versus standard therapy^54^. In this trial, patients were eligible if they received NAC with an anthracycline or taxane (or both) and either had lack of pCR or had tumor CR but residual tumor in lymph nodes, defined by a non-ypT0 or non-ypN0 result or both (Table 1). The capecitabine adjuvant therapy was performed in combination with post-lumpectomy radiation and concurrent hormonal therapy for those patients who had ER-positive disease. Notably, 68% of patients in the CREATE-X trial had ER-positive/HER2-negative breast cancer, and this group received adjuvant hormonal therapy in combination with capecitabine.

**Table 1.** Neoadjuvant chemotherapy (NAC) trials that are practice-changing.

	CREATE-X Trial Masuda *et al*. (2017)^54^	Katherine Trial von Minckwitz *et al*. (2019)^58^
Number of patients randomized	910	1486
Breast cancer subtype	HER2-negative	HER2-positive
Neoadjuvant therapy	Anthracycline, taxane, or both	Taxane (with or without anthracycline) and trastuzumab
Eligibility*	No complete response (CR) on pathological assessment or a CR with positive lymph nodes	Residual invasive disease in the breast or axilla at surgery
Adjuvant therapy	Capecitabine vs. nothing; standard endocrine therapy for hormone receptor positive cancer	Trastuzumab emtansine vs. trastuzumab; standard endocrine therapy for hormone receptor positive cancer
Disease-free survival	At 5 years: 74.1% vs. 67.6%	At 3 years: 88.3% vs. 77.0%
Overall survival	At 5 years: 89.2% vs. 83.6%	To be reported.

*Eligibility assessed after neoadjuvant therapy.

Adjuvant capecitabine was safe and effective and was associated with improvement of DFS and OS in the whole population, but most of the effect was observed in patients with TNBC^54^. For the entire group of patients, there was an improvement in DFS and OS, and this was also true in the subset of patients with TNBC where there was a 5-year DFS (alive and free from recurrence or second cancer) of 69.8% in the capecitabine group versus 56.1% in the control group (HR for recurrence, second cancer, or death 0.58, 95% CI 0.39–0.87) and a 5-year OS rate of 78.8% versus 70.3% (HR for death 0.52, 95% CI 0.30–0.90)^54^. In a planned subset analysis, patients with hormone receptor–positive breast cancer did not have a statistically significant benefit from adjuvant capecitabine; the DFS rates were 76.4% in the capecitabine group and 73.4% in the control group (HR for recurrence, second cancer, or death 0.81, 95% CI 0.55–1.17), and the OS rates were 93.4% and 94.0% (HR 0.73, 95% CI 0.38–1.40)^54^. These results indicate that most patients with TNBC should be offered NAC, not because sequencing chemotherapy before surgery results in better outcomes, but because NAC may allow the acquisition of actionable information on pCR status that may inform a recommendation for additional adjuvant therapy if the outcome is less than a pCR.

In the CREATE-X trial, patients with hormone receptor–positive breast cancer were allowed concomitant adjuvant hormonal therapy while on capecitabine, a departure from the usual approach of not combining hormonal therapy and chemotherapy, and this represents a limitation of the study. Owing to the concern that hormonal therapy potentially protects cancer cells from chemotherapy, hormonal therapy has not been commonly combined with chemotherapy in breast cancer trials^59^. It is possible that the lack of a significant effect of adjuvant capecitabine in the patients with hormone receptor–positive tumors in the CREATE-X trial was related to concurrent hormonal therapy, but other explanations are also possible.

In a different approach, capecitabine was tested as maintenance therapy for TNBC patients who had completed standard therapy of NAC, surgery, and radiation therapy in the phase 3 randomized trial SYSUCC-001, presented at ASCO 2020. This trial studied the use of 1 year of metronomic capecitabine as maintenance therapy for patients with stage 1b to IIIc TNBC^60^. Maintenance therapy with metronomic capecitabine versus observation showed 5-year DFS of 83% versus 73% (HR 0.63, 95% CI 0.42–0.96, *P* = 0.027). In contrast to CREATE-X, this trial was not based on pCR status after neoadjuvant therapy. This study reinforces the potential value of capecitabine in adjuvant and maintenance treatment strategies for TNBC.

Adjuvant capecitabine was also tested in early TNBC in the GEICAM/2003-11_CIBOMA/2004-01 trial following standard neo-/adjuvant chemotherapy^61^. In this study, patients with operable TNBC 1 cm or greater, regardless of nodal status, were randomized to eight 3-week cycles of adjuvant capcitabine or observation after completion of adjuvant or neo-adjuvant chemotherapy consisting of anthracycline and/or taxane treatment. Patients were pre-stratified using a number of criteria. Median follow up was 7.3 years. DFS was not prolonged with capecitabine vs. observation (HR, 0.82; 95% CI, 0.63–1.06; *P* = 0.136). In a preplanned subset analysis, nonbasal TNBC patients appeared to have some benefit of capecitabine vs. basal with a DFS HR of 0.53 vs. 0.94 (interaction test *P* = 0.0694) and an HR for OS of 0.42 vs. 1.23 for basal phenotype (interaction test *P* = 0.0052). The conclusion of this study was that overall, there was failure of added capecitabine to show an improvement in DFS. The GEICAM/2003-11 trial cannot be directly compared with CREATE-X because the patient populations were different and CREATE-X tested capecitabine in patients with more advanced disease that was known to be resistant to chemotherapy. The CREATE-X approach remains an accepted standard of care^62^.

As an alternative to adjuvant capecitabine for TNBC, other approaches are being developed for failure to achieve a pCR in response to NAC. For patients with TNBC and residual disease after NAC, the SWOG 1418 trial tests pembrolizumab adjuvant therapy versus none for at least 1 cm of residual invasive cancer or positive lymph nodes (ypN1mi, ypN1-3) after NAC (NCT02954874).

## Rationale for neoadjuvant therapy as a standard of care for patients with HER2-positive breast cancer

For patients who have received neoadjuvant treatment for early-stage HER2-positive breast cancer, pCR status after HER2-directed neoadjuvant therapy is also informative for optimization of adjuvant therapy. The KATHERINE trial (NCT01772472) was a phase 3 trial for patients with HER2-positive breast cancer and residual disease in the breast and in the axilla or not after NAC with a taxane (with or without an anthracycline) and trastuzumab (Table 1), comparing efficacy of adjuvant T-DM1 versus trastuzumab^58^. Patients meeting eligibility for this trial were required to have T1 to T4, N0 to N3, and M0 clinical stage before neoadjuvant treatment (excluding T1aN0 and T1bN0) and residual invasive disease detected by pathology in the surgical specimen of the breast or axillary lymph nodes after neoadjuvant treatment. This open-label trial randomly assigned patients to adjuvant T-DM1 or trastuzumab for 14 cycles (3 weeks each)^58^. The primary endpoint was invasive DFS. At the 3-year interim analysis, patients who received T-DM1 had a 50% lower incidence of recurrent breast cancer or death (95% CI 0.39–0.64; *P* <0.001)^58^. The estimated rates of freedom from invasive disease were 88.3% in patients who received T-DM1 and 77.0% in patients who received trastuzumab. In both arms, about 72% of patients had hormone receptor–positive breast cancer. Notably, in contrast to trials of neoadjuvant T-DM1, including I-SPY 2, adjuvant T-DM1 in the KATHERINE trial was given concurrently with hormonal therapy to all patients with hormone receptor–positive tumors. It remains undetermined whether combining hormonal therapy with T-DM1 affects outcomes. As in CREATE-X, there is a question of whether hormonal therapy reduces the effectiveness of chemotherapy or an ADC.

In summary, for patients meeting KATHERINE trial inclusion criteria who lacked a pCR of the primary tumor or lymph nodes after neoadjuvant HER2-directed therapy, T-DM1 adjuvant therapy was associated with improved invasive DFS with OS to be reported^58^.

For patients attaining a pCR, continuation of the HER2-targeted regimen that led to the pCR makes sense as adjuvant therapy, while failure to attain pCR would prompt a change of the adjuvant regimen to T-DM1. Therefore, determination of pCR status can inform adjuvant therapy to improve outcomes for patients with HER2-positive tumors where neoadjuvant therapy has failed to yield a pCR. Furthermore, effectiveness of T-DM1 as adjuvant therapy after failure to attain a pCR in the HER2-positive subtype has led to novel research strategies that seek to compare neoadjuvant regimens that de-escalate therapy in the HER2 subtype and could include regimens that delete anthracycline. One neoadjuvant trial that tested deletion of anthracycline on some arms was the TRYPHAENA trial, which investigated the role of neoadjuvant pertuzumab in a randomized phase 2 design^57,63^. This trial showed low rates of systolic ventricular dysfunction with anthracycline free therapy.

Notably, patients with clinical stage T1aN0 or T1bN0 HER2-positive tumors may not need neoadjuvant therapy and may be candidates for upfront surgical management followed by trastuzumab/paclitaxel, as established by the adjuvant phase 2 APT trial for node-negative HER2-positive breast cancer. The APT trial tested 12 weeks of weekly paclitaxel and trastuzumab followed by trastuzumab to complete one year of HER2-directed therapy for node-negative patients with tumors of not more than 3.0 cm, the majority being T1c or smaller^64,65^. This trial, with a median follow-up of 4.0 years, showed a 3-year invasive DFS rate of 98.7% (CI 97.6 to 99.8). The 7-year follow-up showed a DFS of 93% (CI 90.4–96.2) and an OS of 95% (CI 92.4–97.7). Of note, the absence of anthracycline in the APT trial was also associated with low cardiac toxicity compared with historical comparisons and this result points toward de-escalation strategies described below^66^.

Whether concurrent neoadjuvant hormonal therapy contributes to pCR rates in hormone receptor–positive/HER2-positive breast cancer is being studied in the NRG Oncology/NSABP B-52 trial (NCT02003209)^67^. Patients with hormone receptor– and HER2-positive breast cancer were randomly assigned to receive docetaxel/carboplatin/trastuzumab/pertuzumab (TCHP) with or without estrogen deprivation therapy. Although the two approaches were not antagonistic, they may be less than additive; pCR (breast and nodes) rates were 40.9% for TCHP and 46.1% for TCHP and estrogen deprivation (*P* = 0.057)^67^.

## De-escalation of NAC for HER2-positive breast cancer

De-escalation of NAC is a clinical trial strategy to maintain or improve efficacy while reducing long-term toxicity. De-escalation in HER2-positive breast cancer relies on two principles to inform trial design. First, the EFS and DRFS benefits associated with pCR may be agnostic to treatment type so that, if there are many pathways to the pCR goal, the least toxic pathway is favored^45^. Second, there is latitude to test less toxic approaches to reach pCR, because there is the option of rescue by giving T-DM1 or, depending on the neoadjuvant regimen, chemotherapy after surgery. The NeoSPHERE trial studied the efficacy and safety of neoadjuvant pertuzumab and/or trastuzumab plus or minus a taxane in women with early or advanced HER2-positive breast cancer and tested four arms: (A) trastuzumab plus docetaxel, (B) pertuzumab and trastuzumab plus docetaxel, (C) pertuzumab and trastuzumab, and (D) pertuzumab plus docetaxel^68^. The pCR rates were 45.8% (95% CI, 36.1–55.7) for arm B vs. 29.0% (95% CI, 20.6–38.5) for arm A (*P* = 0.0141). For the other arms, the pCR rates were 24.0% (95% CI, 15.8–33.7) for arm D and 16.8% (95% CI, 10.3–25.3) for arm C. After surgery, patients received fluorouracil/epirubicin/cyclophosphamide (FEC) chemotherapy and completed a year of trastuzumab, but based on what is now known about the association between pCR and EFS or DRFS, it is possible that FEC could have been omitted for patients exhibiting pCR without sacrificing EFS or DRFS. Concepts of de-escalation are being tested prospectively in trials such as the DAPHNe trial (NCT03716180), which studies de-escalation of adjuvant therapy to antibodies alone after attainment of pCR with paclitaxel/trastuzumab/pertuzumab. Whereas lower-dose chemotherapy may result in resistant tumor populations, targeted therapies may overcome this problem in certain patients as evidenced by the rare, but notable, pCR observed for some NeoSPHERE patients who received antibodies alone. As yet, we do not know how to identify these uncommon patients who may have a pCR with HER2-targeted therapy alone^68^. Biomarkers may help identify patients who might be predicted to have a pCR response with de-escalated therapy. Notably, high HER2 membrane protein expression was associated with sensitivity to pertuzumab whereas PIK3CA exon 9 mutation was associated with lack of sensitivity to HER2 monoclonal antibody treatment^69^.

Another neoadjuvant trial that addressed the deletion of anthracyclines was the TRAIN-2 study, which randomly assigned patients with stage II or III HER2-positive breast cancer to NAC with or without anthracyclines in the presence of dual HER2 blockade with trastuzumab and pertuzumab^70^. In the anthracycline group, pCR was achieved in 67% (95% CI 60%–73%) of patients; in the non-anthracycline group, pCR was achieved in 68% (95% CI 61%–74%) of patients. In this trial, the non-anthracycline group had a lower incidence of febrile neutropenia but long-term cardiac outcomes remain to be reported. The TRAIN-2 trial argues that de-escalation by deletion of anthracycline is feasible in the neoadjuvant setting for HER2-positive patients; this is consistent with the prior adjuvant BCIRG-006 trial, where non-anthracycline chemotherapy was associated with similar efficacy as anthracycline-containing therapy with lower incidence of cardiac toxicity and leukemia^71^.

It is possible that future neoadjuvant studies for HER2-positive breast cancer will test deletion of neoadjuvant anthracyclines for patients exhibiting an early response as measured by MRI or positron emission tomography (PET). A rapid and deep reduction of the apparent diffusion coefficient (ΔADC) of the primary breast tumor measured by MRI (ACRIN 6698)^4^ predicts pathologic response across combined subgroups. In the ACRIN 6698 trial, ΔADC was moderately predictive of pCR at mid-treatment/12 weeks of weekly paclitaxel (receiver operator curve [ROC] area under the curve [AUC] = 0.60, 95% CI 0.52–0.68; *P* = 0.017), although with subtype analysis, ΔADC was only predictive in the hormone receptor-positive/HER2-negative subgroup^4^. Although the MRI strategy was not used to test deletion of anthracycline, this approach could be tested in future studies to determine whether MRI can facilitate decision making for deletion of anthracycline that might follow 12 weeks of weekly paclitaxel. Similarly, in the TBCRC026 trial a PET scan approach was used to test a chemotherapy-free neoadjuvant regimen of pertuzumab and trastuzumab for ER-negative/HER2-positive breast cancer. The decrease of the maximum standardized uptake value of the primary tumor corrected for lean body mass (SULmax) was moderately predictive of pCR in hormone receptor negative/HER2-positive breast cancer (ROC AUC 0.76, 90% CI 0.67–0.85)^72^. For patients who received a pCR versus not, a SULmax reduction by cycle 1 day 15 of at least 40% was more prevalent (86% versus 46%; *P* <0.001)^72^.

## Rationale for NAC for high-risk ER-positive/HER2-negative breast cancer

Risk stratification is important for determining whether to offer NAC in the ER-positive/HER2-negative subtype. Risk may be determined using genomic assays such as the MammaPrint, 21 gene recurrence score (RS), or a functional assay of Ki67 in combination with clinical parameters (*see Rationale for neoadjuvant endocrine therapy below*). In the I-SPY 2 trial, risk stratification by the 70-gene MammaPrint test is used to identify high-risk patients who are candidates for NAC^44^. Notably, in the I-SPY 2 trial, the ER-positive/HER2-negative control arm had a pCR rate of only 13%^44^. This modest pCR rate is consistent with other trials testing the impact of NAC on pCR in this subtype^73^. In the MammaPrint-high ER-positive/HER2-negative signature, the PD-1 antibody pembrolizumab in combination with weekly paclitaxel was the first experimental agent that resulted in an improvement in the pCR rate (from 13 to 30%)^44^. Comparison of the effectiveness of neoadjuvant pembrolizumab versus placebo for improving the pCR rate in ER-positive/HER2-negative breast cancer is being performed in the phase 3 KEYNOTE-756 trial (NCT03725059). Further supporting a role for immune checkpoint neoadjuvant therapy in high-risk ER-positive/HER2-negative breast cancer was the graduation of the PD-L1 inhibitor antibody durvalumab and the PARP inhibitor olaparib in combination with paclitaxel in the ER-positive/HER2-negative signature, driven by the MammaPrint ultrahigh (MP2) subgroup. In the MP2 subgroup, durvalumab/olaparib/paclitaxel combination exhibited a pCR of 64% versus 22% for the control chemotherapy arm^50^.

## Rationale for neoadjuvant endocrine therapy for ER-positive/HER2-negative breast cancer: a de-escalation strategy

A number of key questions have been addressed by trials of neoadjuvant endocrine therapy (NET) as an alternative to NAC—a form of de-escalation. These studies were based, in part, on the hypothesis that some hormone receptor–positive breast cancers may exhibit equal or greater response to endocrine therapy as chemotherapy^74^. One question addressed by NET trials was to identify the optimal type of endocrine therapy in postmenopausal patients: tamoxifen, aromatase inhibitor, fulvestrant, or a combination of AI and fulvestrant. This goal may be accomplished by comparing the endocrine-sensitive disease rate between endocrine regimens (ESDR; see below). Another question was whether reduction of tumor size can be accomplished by NET, thereby allowing breast-conserving surgery in patients for whom mastectomy would otherwise have been performed^13^. Yet another question was whether information can be gained to elucidate the biological mechanisms of tumor response to endocrine therapy. Furthermore, it may be asked whether there are surrogate endpoints for recurrence free survival (RFS) that identify patients who will do well without adjuvant chemotherapy, thus contributing to de-escalation strategies. If successful, the promise of NET was to increase the chance of breast conservation with lower treatment morbidity and also increase the chance of helping patients who are poor candidates for chemotherapy because of either comorbidities or a desire not to have chemotherapy^74^. The pivot on which this approach rests is the use of a functional assay (PEPI or mPEPI; see below) to assess response to hormonal therapy and then triage to chemotherapy (if necessary) in contrast to the use of a genomic assay to screen or pre-stratify patients.

To begin investigation of the above questions, a study of NET was performed in postmenopausal women to compare neoadjuvant letrozole versus tamoxifen in stage II and III hormone receptor-positive breast cancer with endpoints of response rate (by ultrasound) and whether breast-conserving surgery could be performed^13^. The response rate to letrozole was higher than tamoxifen (60% versus 41%; *P* = 0.004), and the rate of breast-conserving surgery was also higher with letrozole (48% versus 36%; *P* = 0.036). A second trial, P024, also compared preoperative letrozole with tamoxifen in postmenopausal patients with hormone receptor–positive breast cancer^75^. This randomized phase 3 trial compared 4 months of neoadjuvant therapy with letrozole or tamoxifen in patients not amenable to breast-conserving surgery and with T2 or higher T stage, including N1 and N2 patients. The response rate (by palpation) was higher with letrozole (55% versus 36%; *P* <0.001), and the rate of breast-conserving surgery was also higher with letrozole (45% versus 35%; *P* = 0.022)^75^. Comparing letrozole and tamoxifen, secondary endpoints met included ultrasound response rates (35% versus 25%; *P* = 0.042), mammographic response (34 versus 16%; *P* <0.001), and breast-conserving surgery (45% versus 35%; *P* = 0.022)^75^. Notably, in a recent meta-analysis of NET examining 20 randomized clinical trials with a total of 3490 patients, NET was associated with clinical and radiographic response rates and breast-conserving surgery rates similar to those of neoadjuvant combination chemotherapy, even as monotherapy, and toxicity was lower^76^. However, the rates of pCR with NEC were less than 10%. Although pCR in response to endocrine neoadjuvant therapy is prognostic of RFS, it is also uncommon^77^. Reduction of Ki67 in response to NET is also prognostic and predictive of pCR^77–79^. The optimal duration of neoadjuvant aromatase inhibitor therapy appeared to be between 4 and 6 months in one study and between 4 and 8 months in another^80,81^. In terms of biological correlates, neoadjuvant letrozole was associated with a reduction of gene expression associated with cell cycle progression, inhibition of apoptosis, and tissue invasion^82^.

The utility of pCR as an endpoint for NET is controversial and better functional endpoints are needed for lower risk hormone receptor-positive/HER2-negative breast cancer, where pCR rates are low. The pathway to develop better functional biomarkers with clinical utility for assessment of response to NET was made possible with further follow-up of the P024 trial^74,75,83^. Once there was a median follow-up of more than 60 months, it was possible to use outcomes data to develop a prognostic index called the Preoperative Endocrine Prognostic Index (PEPI) score^84^. The PEPI Cox proportional hazards model uses change of pathologic T stage, lymph node status, the Ki67 proliferative index, and the level of ER expression (Allred score) after 4 months of preoperative endocrine therapy to predict RFS and breast cancer specific survival (BCSS)^84^. A PEPI score of 0 is defined as pT1/T2, pN0, Ki67 <2.7%, ER positive (Allred score 3-8) after NET with an AI or tamoxifen and identified patients at low risk for recurrence without adjuvant chemotherapy in prior trials^84,85^. The PEPI score was developed with the P024 trial data and it was demonstrated that its individual components were independently associated with both RFS and BCSS^84^. The PEPI score was validated in the IMPACT trial^86^, an independent study of postmenopausal women with ER-positive, invasive, nonmetastatic, and operable or locally advanced and potentially operable breast cancer, which compared treatment with anastrozole, tamoxifen, or the combination for 3 months before surgery^79,86,87^. A higher Ki67 at 2 weeks of endocrine therapy was found to be prognostic of lower RFS in the IMPACT trial (*P* = 0.004)^79^. With a median follow-up of about 60 months in the IMPACT trial^86^, there were no relapses in patients with stage 0 or I disease and a PEPI score of 0, suggesting that this group of patients is unlikely to benefit from chemotherapy^84^.

The PEPI score was also tested in the ACOSOG Z1031 trial to determine whether it could be used to assist decisions for neoadjuvant endocrine therapy vs. chemotherapy and identify patients with low risk of recurrence. The Z1031 trial tested 16 to 18 weeks of aromatase inhibitor in postmenopausal stage II or III ER-positive patients^85^. Patients with a Ki67 of >10% at 4 weeks of AI were declared endocrine therapy resistant and went on to neoadjuvant chemotherapy^85^. Patients with a PEPI score of 0 exhibited a 5-year risk of relapse of 3.6% without chemotherapy^85^, and confirmation is being sought in the ALTERNATE trial (see below)^88^.

In the ALTERNATE trial, comparison is made of the effects of AI, the selective estrogen receptor down regulator (SERD) fulvestrant, and their combination. Because SERDs downregulate the estrogen receptor, it was necessary to have a modified PEPI score that did not depend on the Allred ER score. A modified PEPI score, the mPEPI score, which eliminates the Allred ER score was developed and validated. The mPEPI score defined as pT1/2, pN0, Ki67 ≤ 2.7%, was found to have similar utility to the PEPI score in patients treated with selective estrogen receptor down regulator (SERD) therapy fulvestrant or AI^89^. Prospective validation of the PEPI and mPEPI 0 biomarker was then tested as a surrogate for RFS in the ALTERNATE trial (Alternate Approaches for Clinical Stage II or II Estrogen Receptor Positive Breast Cancer Neoadjuvant Treatment in Postmenopausal Women: A Phase 3 Study; NCT01953588), which tested whether neoadjuvant fulvestrant or fulvestrant in combination with anastrozole is more effective than anastrozole alone in ER-positive/HER2-negative patients^88^. The ALTERNATE trial was designed to test prospectively, for its first primary endpoint, whether the ESDR or number of patients with an mPEPI score of 0 per number of eligible patients initiating NET with fulvestrant or the combination of fulvestrant and anastrozole is improved relative to anastrozole alone. The patients (n = 1299) were T2, T3, or T4a–c; N0, N1–3, or Nx; and Eastern Cooperative Oncology Group (ECOG) performance score (PS) 0–2. There was 84% power to detect an at least 10% increase in ESDR (in the fulvestrant arms) versus anastrozole alone. The second primary endpoint is to determine whether the 5-year RFS rate for patients with an mPEPI score of 0 and treated adjuvantly with anastrozole without chemotherapy is at least 95% (to be reported around 2025). The duration of NET in the ALTERNATE trial was 24 weeks. Resistance to endocrine therapy was defined by Ki67 of more than 10% after 2 to 4 weeks of NET, based on prior studies^85^, progressive disease documented at any time during NET, pT3/4 or pN1–3 at surgery, or Ki67 of more than 2.7% at surgery (that is, non-0 mPEPI score) or discontinuation of neoadjuvant therapy for any reason. Patients with Ki67 of more than 10% at week 4 or 12 (22.0% of patients) were triaged to NAC. Patients with a non-0 mPEPI score (49.7% of patients) were offered adjuvant chemotherapy followed by hormonal therapy of the physician’s choice. Presented at ASCO 2020, neither fulvestrant nor fulvestrant and anastrozole improved ESDR compared with anastrozole alone in postmenopausal women with locally advanced ER-positive/HER2-negative breast cancer^90^. ESDR for anastrozole was 18.5% (95% CI: 15.1–22.7%), 22.7% for fulvestrant (95%CI: 18.9–27.0%), and 20.5% for the combination (95% CI: 16.8–24.65%). RFS data remain to be reported and will tell us prospectively whether the mPEPI score can predict long-term RFS^90^. There were several key findings. Very few patients had disease progression before surgery. If the baseline Ki67 was not more than 10%, it remained low. If the Ki67 was greater than 10%, about two thirds of the patients showed Ki67 reduction. The ALTERNATE trial also showed feasibility of week 4 biopsies across a trials network. We await the 5-year RFS value for the patients with an mPEPI score of 0, a measure of breast cancer-free interval.

Prospective validation of the PEPI 0 and mPEPI 0 biomarker as a surrogate for 5-year RFS in the ALTERNATE trial would allow comparison of different NET strategies and agents and identify patients who do not need chemotherapy- a de-escalation strategy. Thus, neoadjuvant hormonal therapy is also a pathway to de-escalation because although higher-risk patients in the ALTERNATE trial were offered postoperative chemotherapy, many of these patients who received neoadjuvant hormonal therapy were able to avoid postoperative adjuvant chemotherapy. Therefore, in the ALTERNATE trial, postoperative chemotherapy served as a backstop for those patients who were candidates for it, whereas lower-risk patients avoided it^90^.

A different approach is to use a genomic assay rather than a functional assay to predict which patients may have a higher response rate with NET. Notably, the 21-gene Recurrence Score (RS) (Oncotype DX; Genomic Health, now part of Exact Sciences, Madison, WI, USA) assay has been validated to predict clinical response of ER-positive/HER2-negative breast cancer to neoadjuvant hormonal therapy using letrozole in the prospective TransNEOS study (n = 295 patients)^91^. Response was measured by imaging with complete response (CR) or partial response (PR) or both being compared with stable disease (SD) or progressive disease (PD). Clinical response rates for RS less than 18, 18–30, and 31 or greater were 54%, 42%, and 22%, respectively. In a multivariate analysis, a higher proportion of patients with RS of less than 18 exhibited CR/PR compared with patients with RS of at least 31 (*P* <0.001). Of patients with RS of 31 or greater, 17% had progression on letrozole. In summary, the RS may allow de-escalation of neoadjuvant therapy for lower-risk ER-positive/HER2-negative breast cancer patients. In contrast, the PEPI/mPEPI score paradigm uses a functional approach to determine which patients may do well with endocrine therapy alone and is being tested for prognostic value. While MammaPrint can be used to estimate benefit from chemotherapy, its role in neoadjuvant endocrine therapy remains to be determined. In the Neoadjuvant Breast Registry Symphony Trial (NBRST), a prospective registry trial (n = 426 patients), there were 59 patients who were MammaPrint low risk and Luminal A^92^. Of these patients, 20 patients received NET and they exhibited a 65% PR rate, while 37 received NAC^92^. These observation suggests that the RS and possibly the MammaPrint score may be useful to identify patients who are candidates for NET trials comparing agents and regimens. Further trials could address the question of whether RS or MammaPrint predict an effect of NET on 5-year RFS.

## Addition of cyclin-dependent kinase inhibitors to neoadjuvant endocrine therapy

The activity of neoadjuvant hormonal therapy in combination with cyclin-dependent kinase inhibitors (CDKis) (for example, palbociclib, ribociclib, and abemaciclib) is an area of active investigation. The NeoPalAna trial was a study of 50 patients, in whom neoadjuvant palbociclib was tested in combination with anastrozole in clinical stage II or III ER-positive/HER2-negative breast cancer^93^. In this single-arm phase 2 trial, anastrozole monotherapy was administered for 4 weeks followed by the addition of palbociclib (21 days on, 7 days off) for four 28-day cycles, provided that Ki67 was not more than 10%. Anastrozole was continued until surgery and a fifth shorter cycle of palbociclib (10–12 days) was given immediately before surgery. The primary endpoint was complete cell cycle arrest (CCCA), defined as Ki67 of not more than 2.7%. The CCCA rate was 87% on cycle 1 day 15 versus 26% on cycle 1 day 1 (*P* <0.001). No pCRs were observed. Response rates were 80% (90% CI, 68%–90%), 41% (25%–58%), and 52% (35%–68%), by exam, ultrasound, and mammogram, respectively. Resistance was associated with non-luminal subtypes and persistent expression of E2F target genes^93^.

In the randomized phase 2 PALLET trial, the effects of adding palbociclib to neoadjuvant letrozole were studied in postmenopausal women with ER-positive/HER2-negative primary breast cancers of at least 2.0 cm in size^94^. Patients were assigned treatment with either letrozole (14 weeks), letrozole for 2 weeks followed by the addition of palbociclib to 14 weeks, palbociclib for 2 weeks and then palbociclib plus letrozole to 14 weeks, or palbociclib plus letrozole for 14 weeks^94^. Clinical response did not differ with the addition of palbociclib to letrozole (*P* = 0.20; complete response + partial response, 54.3% v 49.5%). Addition of palbociclib resulted in a greater median log-fold change in Ki-67 (-4.1 v -2.2; *P* <0.001). Grade 3 toxicity was increased with the addition of palbociclib to letrozole and this was mostly related to asymptomatic neutropenia. Six patients had a pCR out of 180 patients receiving the palbociclib-plus-letrozole regimen (3.3%).

## Addition of cyclin dependent kinase inhibitors to adjuvant endocrine therapy

Recently, the phase 3 Palbociclib Collaborative Adjuvant Study (PALLAS trial) (press report) and monarchE^95^ adjuvant trials testing different CDKi’s in endocrine receptor–positive/HER2-negative breast cancer have reported results. The PALLAS trial (NCT02513394) investigated a primary endpoint of invasive DFS (IDFS) for patients who received standard endocrine therapy alone or in combination with the CDK4/6 inhibitor palbociclib for 2 years. PALLAS was a multicenter open-label randomized trial that studied patients with stage II or III breast cancer. A preplanned analysis was performed, and the PALLAS trial was stopped because of futility (announced May 29, 2020). The monarchE trial (NCT03155997) also measured a primary endpoint of IDFS for patients who received standard endocrine therapy alone or in combination with the CDK4/6 inhibitor abemaciclib for 2 years. monarchE is a multicenter open-label randomized trial that studies a population of patients at high risk for recurrence with four or more pathologically positive lymph nodes or one to three lymph nodes and high-risk features such as primary tumor of at least 5 cm, grade 3 tumor, or Ki67 of at least 20%. monarchE showed a significant improvement in IDFS with abemaciclib and endocrine therapy versus endocrine therapy alone (*P* = 0.01; HR 0.75, 95% CI 0.60–0.93) with respective IDFS rates of 92.2% versus 88.7% at 2 years^95^. The monarchE paper and the PALLAS trial press release indicate a difference between CDKi types in the adjuvant setting. Whether there are differences between CDKi’s in the neoadjuvant setting remains to be seen.

In summary, the roles for NET include downstaging of ER-positive/HER2-negative breast cancer before surgery, identifying tumors that may not require chemotherapy, and providing insights about the biology of this breast cancer subtype. We await determination of whether biomarkers of NET tumor response correlate with RFS. Another potential role of NET, which has recently emerged, is to delay surgery for some patients with the ER-positive/HER2-negative subtype of breast cancer during the COVID-19 pandemic, as described below.

## Relevance of neoadjuvant hormonal therapy in the COVID-19 pandemic

During the COVID-19 pandemic, there is increased need for flexibility of health-care resource utilization while prioritizing the goal of cure for our patients^96^. Ideally, decisions to use neoadjuvant therapy as opposed to upfront surgery are made by a multidisciplinary team consisting of the surgical oncologist, medical oncologist, radiation oncologist, and medical geneticist^96^. Before, COVID-19 patients with smaller tumors (≤1 cm) who were clinically node-negative were good candidates for upfront surgery, regardless of histology, and they remain so since COVID-19. Nonetheless, neoadjuvant therapy is an option for patients with smaller node-negative tumors, where surgery followed by adjuvant chemotherapy would have been appropriate pre-COVID. Patients who would have been candidates for NAC before COVID-19—including patients with TNBC, HER2-positive, and genomic high-risk (MammaPrint)/clinical high-risk hormone receptor–positive/HER2 negative breast cancer—remain so. A difference since COVID-19 is that patients with stage I to III hormone receptor–positive/HER2-negative tumors that are genomic low-risk (MammaPrint)^25^ may be treated with NET to delay surgery while potentially promoting breast conservation in some cases. Similarly, selected node negative patients over age 50 with a 21-gene RS of less than 26 may be candidates for neoadjuvant hormonal therapy^96,97^. With NET or NAC there is risk of disease progression and appropriate monitoring with clinical exam, ultrasound, and other imaging are important to assess response to NET over time. Furthermore, some hormone receptor positive/HER2-negative breast cancers that appear clinical low risk by pre-operative staging and are genomic low risk by pre-operative biopsy may still be recommended to have adjuvant chemotherapy depending on the outcome of surgical staging after NET and this possible outcome needs to be discussed with patients at the outset of NET. Once the pandemic is past, the optimal use of the RS or MammaPrint in combination with clinical risk assessment to direct NET and integration with ultrasound, MRI, and biopsy results requires further clinical trials development^91,92^.

## Highlights and future directions

An important future direction will be to determine how neoadjuvant/adjuvant therapy can be further developed to improve outcomes in high-risk ER-positive/HER2-negative breast cancer now that we have learned that neoadjuvant immune checkpoint therapy improves pCR rates in high-risk and ultra-high risk ER-positive/HER2-negative MammaPrint subgroups^44^. Although pCR was achievable in only 13% of patients with the MammaPrint high-risk ER-positive/HER2-negative signature treated with the standard-of-care arm in the I-SPY 2 trial, the PD-1 antibody pembrolizumab resulted in a 30% pCR rate in this signature^44^. Indeed, pembrolizumab was the first agent of the initial nine tested that showed an improvement of pCR rates in the ER-positive/HER2-negative signature. This result was recently followed by the graduation of the PD-L1 antibody durvalumab in combination with olaparib and paclitaxel in the MammaPrint ultrahigh (MP2) ER-positive/HER2-negative signature, where a pCR rate of 64% was observed relative to 22% for control^50^. In TNBC, targeting the PD-1/PD-L1 checkpoint with pembrolizumab increased pCR rates from 22 to 60%^44^ and with durvalumab/olaparib/paclitaxel from 27 to 47%^50^. Notably, PD-1 is a marker of effector T-cell exhaustion^98^, and the I-SPY 2 trial results suggest that targeting T-cell exhaustion markers may be a very productive approach in hormone receptor–positive/HER2-negative breast cancer and TNBC. Lag-3, Tim-3, and TIGIT are other markers of T-cell exhaustion^98^ for which inhibitory antibodies are being developed. Combination therapies to overcome T-cell exhaustion are in development. A novel immune checkpoint strategy involving the combination of a Lag-3 antibody REGN3767^99^ and cemiplimab, a humanized hinge stabilized PD-1 antibody^100^, is being developed in the I-SPY 2 trial. We await with great interest the results of new strategies to overcome T-cell exhaustion.

Another direction for progress will come from ongoing development of more effective strategies for de-escalation of therapy, particularly through measurement of pCR^45^ and MRI biomarkers^4^ in NAC. For example, improvements in HER2-targeted therapy may result in improved responses, measured by reduction of functional tumor volume and biopsy, such that anthracyclines may be deleted from neoadjuvant platform trial regimens that currently contain anthracyclines. Further progress will likely come from the use of functional biomarkers such as the mPEPI and PEPI score^90^, and genomic assays^91^, in NET. The process of answering these questions, through well designed trials will continue to add to the growing body of information on neoadjuvant strategies in breast cancer, where quantitatively measuring the effects of targeted drug therapy has added so much to what we can offer our patients.
